# E-textile tooling: new tools—new culture?

**DOI:** 10.1186/s13731-017-0067-y

**Published:** 2017-04-24

**Authors:** Irene Posch

**Affiliations:** 1 University of Applied Arts Vienna, Vordere Zollamtsstrasse 3, 1030 Wien, Austria; 2 Vienna University of Technology, Argentinierstrasse 8, 1040 Wien, Austria

**Keywords:** Making, Maker culture, Electronics, Crafting, Tools, Diversity, Materiality, Design

## Abstract

**Background:**

The maker movement in recent years has shed light on the blurring boundaries between crafts, creativity, and technology. Tools are a key part of the creation process, shaping both our process of making and the objects we make. They do so through their form and material influence, the matter they can handle, as well as the skills needed to utilize them. Often, tools also evoke stereotypical associations of who is using them and what is being produced with them.

**Findings and Conclusions:**

In the following, I focus on needlework tools and the crafting of electronic textiles. I introduce research into the shape and aesthetics of needlework tools that incorporate the functionality of electronic probes. On a functional level, they can be used to construct pieces of textile crafts as well as to connect and test their electrical functions while making. On a metaphorical level, they allude to a possible alternative realm of creating electronic devices and components. In connecting the skills and aesthetics of textile crafting to electronic objects, we want to spark an exchange between different making cultures and enable diverse approaches for expression.

## Findings

It is an essential aspect of craftsmanship that the quality of the result is not pre-determined, but depends on the judgment, dexterity, and care, all of which the maker exercises while working (Pye 1968). As an artifact and process, craft practices reveal and reflect on the conditions that bring them into being (Adamson 2013). This approach to craft highlights the tools as the means of production that are crucial part in making. Looking at a tool commonly gives a hint at the fabrication space in which it is applied, and this is even more so when seen among a collection of tools belonging to the same domain. The tool’s physical appearance points at properties of the materials it is supposed to handle. The tool’s size, manufacture, and functionality indicate producible forms and frame the possibilities for making. In addition to these functional aspects, historically established stereotypes and gender-specific connotations often surface, suggesting who uses the tool, in what domain it is used, and what artifacts it brings into being (Beaudry 2006).

This research focuses on the field of electronic textiles (e-textiles), an area where textile materiality and electronic capability merge in the creation of textiles that enable electronic and digital functionalities to be embedded in them. Whereas very different in many aspects, textiles and electronics are increasingly seen as potentially complementary rather than mutually exclusive disciplines, spreading into the domains of fashion, product design, material research, and education. Research into how to make textile sensors (e.g., Perner-Wilson et al 2010) and how to ease the integration of computational hardware with textile material (Buechley 2006), among others, have greatly improved the field’s accessibility. A range of kits are available providing specifically designed electronic components that can be sewn onto fabric, as well as conductive and resistive threads for making connections and crafting sensor. All of these enable new groups to engage and contribute to diverse creations (Buechley et al. 2008).

**Fig. 1:**
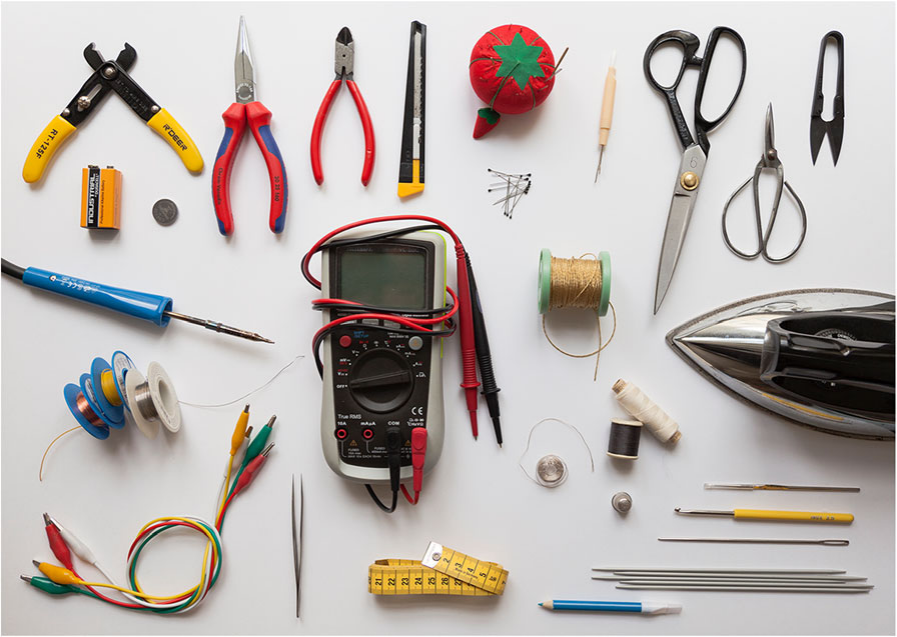
Exemplary collection of tools used in e-textile making, the included tools that traditionally belong to the discipline of textiles or electronics, sometimes also to the jewelry and other related disciplines. In the center a multimeter with a pair of standard multimeter probes. A multimeter is essential to electronic measuring tasks, among others to measure resistances or detect short circuits

While there has been a growing availability of material resources to engage with e-textiles, there has been very little work to date on researching and developing tools specifically to support an e-textile craft: tools that cater to the specific challenges that arise from the merging of distinct disciplines. As a result, practitioners in the field of e-textiles, both professionals and novices, currently rely on a mix of tools from diverse, existing disciplines, a sample being pictured in Fig. 1. However, the handling of soft, often delicate, materials like fabrics and threads demands very different tools than that of wires, metal leads, and fiberglass boards, which constitute the material space for which electronic testing equipment is usually designed. Starting from these basic functional needs, this ongoing research is concerned with bridging this gap in exploring potentials of new tools in the domain of e-textile crafts. This seems especially relevant, as the questions of how to handle and manipulate the material, how to bring it into form, and what aesthetic as well as what functional outcome is producible, is essentially shaped by the tool.

In a stereotypical electronic textile project, the fundamental tools include different needles and scissors for the textile manipulation, a multimeter for electronic measuring tasks, a soldering iron, wire strippers and pliers to handle electronic components and materials. They are a selection of instruments to meet individual goals, but not distinctly attributable to the domain of an electronic textile craft. These tools are adapted to fit the task, or a different way to reach a similar output is found. Borrowing tools from other disciplines can impose challenges to the work though: The tools have not been specifically designed for the task for which they are used; hence, they might not perfectly support the scenario they are applied in. Appropriating existing tools may in many cases fulfill the task just fine. In a less ideal scenario though, their usage may harm the material or the material harm the tool. A seamstress for example would most likely not allow paper being cut with her fabric scissors as it may harm the blades, and one will not be able to achieve a clear cut with paper scissors on fabric in turn. Another challenge might be that the way to hold and apply a tool might interfere with routines otherwise genuine to the craft, and consequently lead to inferior quality of the output. Hence, on a practical level, the question arises whether the availability of more suitable tools could advance an emerging discipline of electronic crafts in terms of enabling better functional and aesthetic explorations. On a metaphorical level, it is interesting to explore if tools genuine to an electronic textile craft might also improve accessibility across diverse making cultures.

## Textile material and electronic function

As briefly discussed before, the disciplines of textiles and electronics seem to be especially far in terms of their material ties and tool-sets used to work these. This makes merging them specifically interesting. It is not only affecting the objects realized but also prevailing assumptions about the coming into being of that object—of the processes, materials, and cultures dominant in creating them (Buechley and Perner-Wilson 2012).

Needlework and textile crafts in general are long established practices, and their tools have undergone many iterations of improvements to reach their current shape (Beaudry 2006). Also, the use of electronically conductive and resistive materials has existed within the domain of textile crafts for a long time. Early descriptions of metal needlecrafts date back to the thirteenth century B.C. (Fürnkranz 2005). Metallic threads, prepared in numerous shapes to fit diverse textile techniques, have been used for ornamental reasons, to produce exquisite garments or textile arts for clerical, royal, and bourgeois clientele. Even though resistive and conductive properties are inherent in their material qualities, they have not been utilized for electronic functionality, and thus, electronic properties of the related tools were not relevant. If crafting for electronic functions, for example, a resistor (Kurbak and Posch 2014) or crocheting a sensor (Perner-Wilson et al 2010), one needs a crochet hook and resistive yarn to actually crochet a functional electronic part, and one simultaneously needs to be informed about the resistance value of the crochet in the making in order to know when the correct value is reached. If crafting an electric circuit, one wants to know that the ends are connected correctly, and ideally already have access to that information while making it to adapt accordingly. The two mentioned routines are standard procedures in electronics, and there are refined tools available for these precise tasks when dealing with hardware electronic components. But existing tools for measuring resistance or detecting electrical connections, as they are designed for a different making context, are mostly not compatible with textile materials. Their probes are for many tasks too big, too rough, or cannot establish good contact on textile, and are even less useable to actually create any textile artifact.

In this section, I briefly describe the research and design of tools for e-textile crafts. Resulting prototypes are currently being tested in real use.^1^ The focus here is illustrating how traditional textile tools and electronic needs can be merged to serve specific e-textile crafts. To start these explorations, we focused on two key electronic routines of measuring resistance values and testing connections, something usually done with a multimeter. The interplay of the material and electronic aspects in these routines is then discussed on the basis of the e-crochet example.

## Exemplary case: the crochet hook multimeter probe

**Fig. 2:**
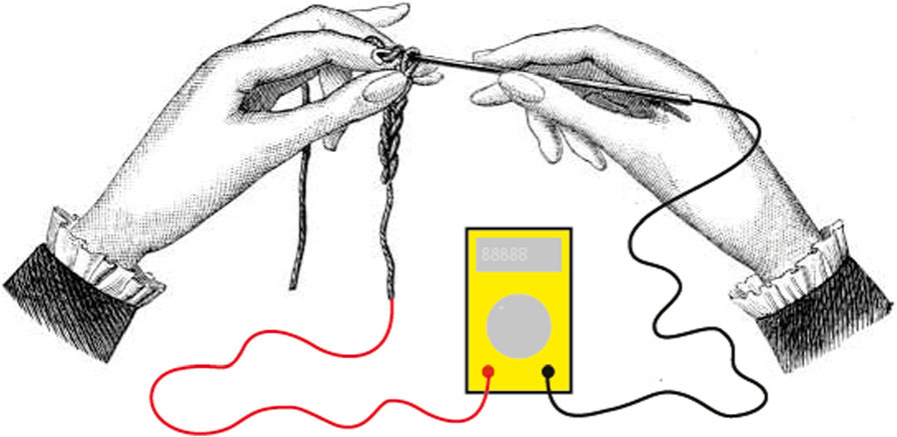
Concept drawing, consisting of an illustration from the Encyclopedia of Needlework by Thèrèse de Dillmont (de Dillmont, 2007), first published in 1886, that shows how to hold and use a crochet hook and a drawing by the author showing the crochet hook and the yarn being electrically connected to a multimeter, an electronic measuring instrument

As a first step towards creating tools that better fit the above described tasks, we created a series of prototypes. We started with looking at the core functionalities of existing tools within the disciplines, the main tasks they are used for, and how they are achieved. Crucial to measuring and testing tasks in electrical engineering are reliable probes made from highly conductive material so the electrical signal can pass lossless. On the other hand, tools used for textile production, such as hooks, needles, rippers, and etc., have fine-modeled tips in different shapes to adequately handle and model the respective textile material. These tips are often made out of metal, presumably for quality reasons. Metal is also highly conductive and hence can be a suitable material for an electronic probe. If the tip of the utensil can be connected to testing or measuring circuits, it could be used concurrently as an electrical probe and a textile tool, as illustrated in Fig. 2.

**Fig. 3:**
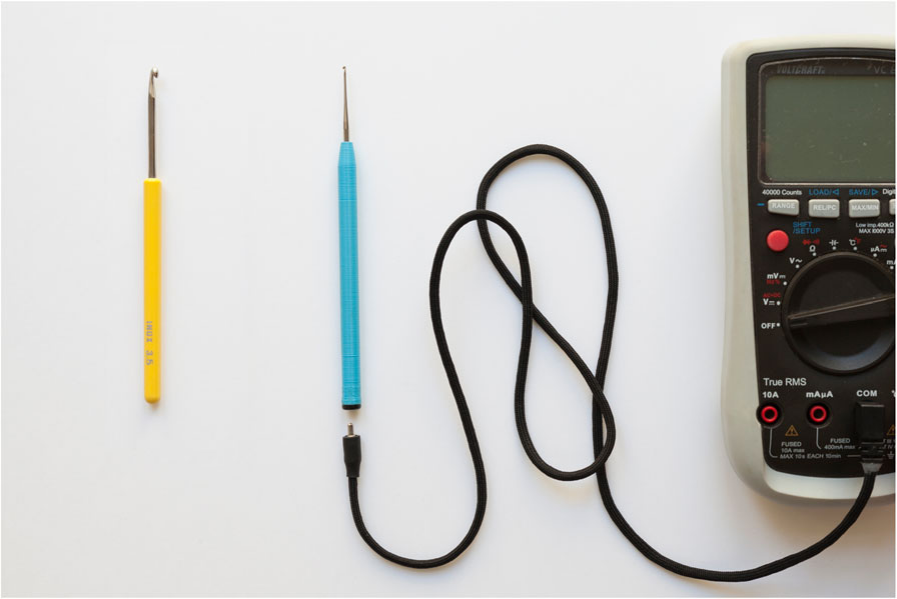
A traditional crochet hook (hook size 3.5) with a yellow handle and a crochet hook multimeter probe (hook size 1.5) with a blue handle and custom-made multimeter cable. The blue crochet hook is connectable to the multimeter cable through a mini banana plug that goes into a mini banana jack at the end of the crochet hook. The multimeter cable on the other side connects to the multimeter

**Fig. 4:**
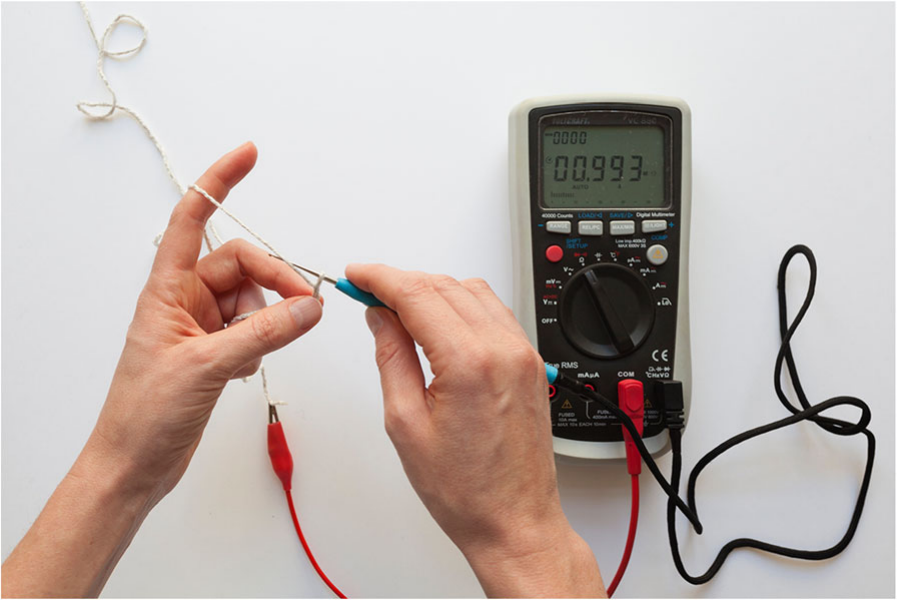
Crochet hook connected to a multimeter to give direct feedback about the resistance value of the crochet in the making

To construct that, we took a crochet hook and removed the tip from the handle and instead put it into a similar-looking but custom 3d-printed mount. The custom mount has a thin hole in the middle along its length to push a wire through. This wire is on one side soldered to the crochet tip and on the other end soldered to a mini banana jack; thus, an electrical conductive connection between the tip of the crochet hook and the end of the crochet hook is established. It now is connectable and usable as an adaptor to external electronic equipment through a mini banana plug. The connectable crochet hook is pictured in Figs. 3 and 4.

To connect the hook, we produced cables that are on the one side compatible with standard multimeter plugs and on the other side feature a mini banana plug to connect to the connectable crochet hook. The cables are also custom constructions, consisting of braided textile cords around a flexible wire that electrically connects its two ends. As opposed to the relative stiffness of common multimeter cables, this soft cable helps avoiding potential harm to a delicate textile artifact or interfering with the crafter’s movements while using the hook. The textile multimeter cable is pictured in Fig. 3, connected to the hook in Fig. 4.

The result is visually and functionally a standard crochet hook but augmented to be a connectable probe to a multimeter, or other electronic testing equipment. It is still the same quality tool when crocheting from a crafting perspective, but it allows crocheting a resistor, or resistive sensor, and simultaneously being informed about the resistance value of the crochet produced, from an electronic perspective. It is a deliberate choice to keep the visual appearance of the probe that of a traditional crochet hook to indicate what materials and creations this tool does address. Assumptions are that such an approach might also help making the electronic functionality as accessible as possible to people experienced in crochet but less experienced in electronics, as they can rely on the existing craft skills and tools they are mastering.

## Critical tooling

In any form of making, control is determined mainly by the “means of production,” who guides their working and how this field of agency is structured (Adamson 2013). The intention driving the creation of our research is to bridge essential qualities when handling textile materials with essential necessities when dealing with electronics. A tool genuinely bridging these fields of crafting and technical expertise—apart from a pure functional solution—might also question currently dominating assumptions about the disciplines involved, as well as about the artifacts emerging from them and the people who bring them into being: What if electronic objects would be the result of textile crafting practices? What if their making and repairing would rely on the skills, materials, and tools native to craft disciplines? How would we interact with such artifacts? Assuming existing skills in knitting, embroidery, and crochet would be beneficial or even essential to electronic making, would this alter the stereotypes we currently have about the field of textile crafts—and would it influence the group of active makers in the field of electronics?

With the example of the connectable crochet hook multimeter probe, we introduced a tool relating to textile making and electronic functionality. In designing tools specific to textile electronic crafts, we want to spark a discussion about a different possible realm of the coming into being of electronic components and devices. In deliberately referencing a century-old craft, and with it its skilled workers, we want to question existing cultures of technology production.

So far, the tool has been successfully tested and used in realizing exemplary work. It has been effective regarding the expectation of providing information about electric properties of a textile artifact in the making, directly integrated into the tactile craft process. In the next step, we plan to continue work on the extension of an e-textile toolkit and its distribution to a broader audience to get a deeper understanding about the practical implications, potentials, and affordances of such tools.

## Footnotes

1 Extensive discussion of the findings is beyond the scope of this paper.
